# Dual effect of the SR proteins ASF/SF2, SC35 and 9G8 on HIV-1 RNA splicing and virion production

**DOI:** 10.1186/1742-4690-2-33

**Published:** 2005-05-22

**Authors:** Sandrine Jacquenet, Didier Decimo, Delphine Muriaux, Jean-Luc Darlix

**Affiliations:** 1 Laboratoire de Médecine et Thérapeutique moléculaire, INSERM CIC9501, 15 rue du Bois de la Champelle, 54500 Vandoeuvre-lès-Nancy, France; 2 LaboRetro, Unité de Virologie Humaine, INSERM #412, Ecole Normale Supérieure de Lyon, IFR 128, 46 allée d'Italie, 69364 Lyon cedex 07, France

## Abstract

In HIV-1 infected cells transcription of the integrated provirus generates the single full length 9 kb viral RNA, a major fraction of which is spliced to produce the single-spliced 4 kb RNAs and the multiple-spliced 2 kb RNAs. These spliced RNAs are the messengers for the Env glycoproteins and the viral regulatory factors. The cellular SR and hnRNP proteins were shown *in vitro *to control alternative splicing by binding *cis*-regulatory elements on the viral RNA. To better understand *in vivo *the role of the SR proteins on HIV-1 genomic RNA splicing and virion production, we used a human cell line expressing high levels of complete HIV-1 and either one of the ASF/SF2, SC35, and 9G8 SR proteins. Results show that over-expressing SR proteins caused a large reduction of genomic RNA and that each SR protein modified the viral 9 kb RNA splicing pattern in a specific mode. In fact, ASF/SF2 increased the level of Vpr RNA while SC35 and 9G8 caused a large increase in Tat RNA. As expected, overexpressing SR proteins caused a strong reduction of total Gag made. However, we observed by immuno-confocal microscopy an accumulation of Gag at the plasma membrane and in intracellular compartments while there is a dramatic reduction of Env protein made in most cells. Due to the negative impact of the SR proteins on the levels of genomic RNA and HIV-1 structural proteins much less virions were produced which retained part of their infectivity. In conclusion, SR proteins can down-regulate the late steps of HIV-1 replication.

## Background

**Figure 1:**
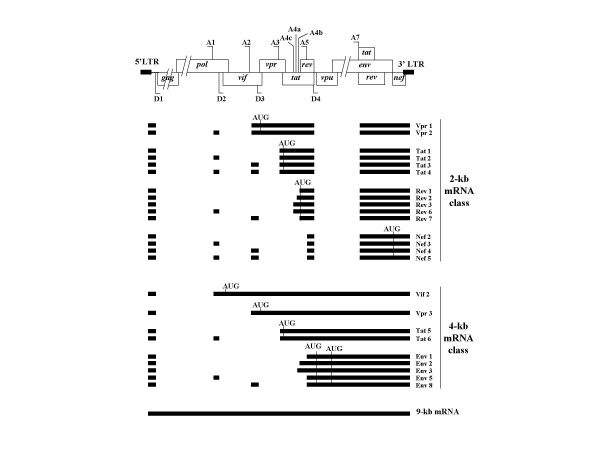
**HIV-1 splicing pattern**. Schematic representation of HIV-1 proviral DNA. Open boxes represent the open reading frames encoding the viral proteins. Black boxes represent exons generated by combination of donor sites (D1 to D4) and acceptor sites (A1 to A7). The viral translation initiator codons are indicated by AUG.

From a genome of only 9000 nt in length, HIV-1 directs the synthesis of 15 proteins essential for its replication and dissemination (for review see ref. [1]). In order to generate mRNAs required for the synthesis of these proteins, HIV-1 uses the cellular splicing machinery. Through alternative splicing of its primary RNA transcript containing 4 donor sites (D1, D2, D3 and D4) and 8 acceptor sites (A1, A2, A3, A4a, A4b, A4c, A5 and A7), more than 30 different mRNAs are generated and divided into three classes of 2 kb, 4 kb and 9 kb in length (Figure 1) [2]. The 2 kb mRNAs are fully spliced and principally encode the regulatory proteins Tat and Rev and accessory proteins Nef and Vpr. The single-spliced 4 kb RNAs are bicistronic and code for the Env glycoproteins and viral factor Vpu, and the unspliced 9 kb RNA serves both as mRNAs for the Gag and Gag-Pol polyproteins as well as pre-genomic RNA for Gag assembly. Rev is crucial because it directs the export of the unspliced and single-spliced mRNAs from the nucleus to the cytoplasm that permits their translation [3,4]. A fine tuning of splicing is then critical to ensure the balance between spliced versus unspliced viral RNAs.

HIV-1 splicing regulation relies on the presence of (i) suboptimal splice sites [5,6], (ii) exonic and intronic *cis*-acting elements [7-15] and (iii) *trans*-acting factors (generally hnRNPs and SR proteins) that mediate their effects by binding these elements [16-19]. SR proteins belong to a conserved family of structurally and functionally related phosphoproteins (for review, ref. [20]). These proteins participate in constitutive splicing by causing stabilizing interactions with components of the splicing machinery and are able to influence the choice of splicing sites in alternative splicing (for review see ref. [20]). The high level of conservation of the splicing pattern in different HIV expressing cells suggests that splicing regulation is critical for efficient virus replication [2,21,22]. Because SR proteins ASF/SF2, SC35, 9G8 and SRp40 have been shown to cause an imbalance in the HIV-1 splicing pattern *in vitro *and *ex vivo *[19,23-26], we investigated the impact of SR protein over-expression on virus production and infectivity in a human cell line expressing infectious HIV-1.

In the present study we show that overexpression of one of the three SR proteins ASF/SF2, SC35 and 9G8 together with HIV-1 strongly affected the full length viral RNA splicing pattern, notably resulting in a strong reduction of the genomic RNA and Env mRNA levels. As a consequence, only small amounts of viral particles were produced which, however, retained part of their infectivity.

## Results

### SR proteins alter the splicing pattern of HIV-1

**Figure 2:**
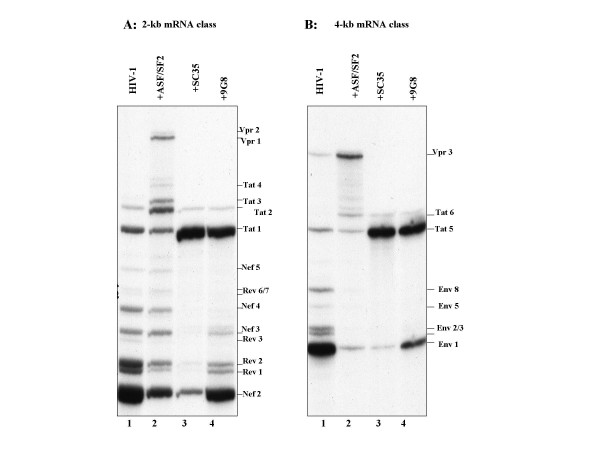
**Regulation of HIV-1 alternative splicing by SR proteins**. Analysis of 2 kb (**A**) and 4 kb (B) mRNAs was performed by RT-PCR using 10 μg of total cellular RNA extracted from 293T cells transfected by HIV-1 pNL4.3 only (lane 1) or together with one SR plasmid (lanes 2–4). Viral mRNAs were identified according to the nomenclature of Purcell and Martin [2].

Human cells (293T) were co-transfected by the calcium phosphate precipitation method with 10 μg of HIV-1 pNL4-3 [27] and 10 μg of irrelevant plasmid pCLacZ (control) or 5–10 μg of one of the SR protein-expression vectors, pXJ41-ASF, pXJ42-PR264 and pXJ42-9G8, encoding respectively ASF/SF2, SC35 and 9G8 proteins [26,28]. Expression of HIV-1 and SR proteins in co-transfected cells was verified by immunoblotting assays (data not shown). We first performed RT-PCR in conditions previously described [2,29] to verify that SR proteins modified HIV-1 splicing pattern as reported elsewhere [26]. Multiple-spliced 2 kb mRNAs isolated from ASF/SF2 over-expressing cells showed that Vpr1, Tat2 and Tat3 were strongly increased as compared with the control (Figures 1, 2A). These observations were confirmed by the analysis of the 4 kb mRNAs where Tat6 and Vpr3 mRNAs became the most represented in these conditions probably at the expense of the Env mRNA which proved to accumulate at a low level (Figure 2B). SC35 and 9G8 overexpression led to similar splicing patterns where Tat1 and Tat5 mRNAs were the most abundant spliced isoforms (Figures 1, 2). In the case of SC35, splicing was almost completely driven towards Tat1 production. Because Tat2 and Tat6 required splicing at site A1 and Vpr1, Vpr3 and Tat3 mRNAs at site A2, we concluded that ASF/SF2 participated in a positive regulation of splicing at sites A1 and A2, while SC35 and 9G8 preferentially enhanced splicing at site A3 necessary for Tat mRNA synthesis (Figure 1). These results are in agreement with those obtained in HeLa cells using a truncated non-infectious HIV-1 DNA construct [26] and showed that SR proteins profoundly changed the HIV-1 splicing pattern. However the effects observed in the present experimental conditions were stronger than with the incomplete HIV-1 DNA construct [26].

To further study the SR-mediated commitment of the full length viral RNA to splicing, that is increasing the ratio of viral spliced *versus *unspliced RNAs, we purified total RNAs from cells expressing HIV-1 and either one of the SR proteins and subjected 10 μg total RNA to Northern blot analysis with an HIV-1 *env*-specific probe. In control HIV-1 cells, 8 % of HIV-1 RNA remained unspliced while this amount was lowered to 0.5% by ASF/SF2 and SC35, and to 1.5% by 9G8. This also caused a decrease of total intracellular viral RNAs by two to five fold (Table 1A). We concluded that SR proteins are general activators of HIV-1 splicing, negatively regulating the steady state level of full length viral RNA.

**Table 1:** Relative levels of the three viral mRNA classes. The amounts of radioactivy in mRNA signals identified by Northern blotting or by slot blotting experiments (see methods) were measured using a Storm scanner. (A) Relative levels of total intracellular viral RNA were determined as the sum of the radioactivity in the 3 signals corresponding to the 2, 4 and 9 kb mRNAs from the same experiments. Levels are expressed as the percentages of total viral RNA in cells transfected with HIV pNL4.3 only used as a reference (100 %) or with HIV-1 pNL4.3 and an SR plasmid. For the same degree of DNA transfection, the percentages of the unspliced and spliced mRNAs were calculated relative to the total viral RNA considered as 100 %. (B) Values of genomic RNA packaged into a standardized amounts of virions (CAp24 ELISA) are reported relative to the virions produced in the absence of SR protein overexpression (100%).

	(A) CELLS			(B) VIRIONS
	
	Total	unspliced	spliced	9 kb
**HIV-1 **(%)	100	8	92	100
+ ASF/SF2 (%)	25	0.5	24.5	24
+ SC35 (%)	20	0.5	19.5	25
+ 9G8 (%)	51	1.5	49.5	37

### Alterations of HIV-1 splicing pattern by SR proteins modify viral protein synthesis

The profound modifications of the HIV-1 splicing pattern by overexpression of one of the SR proteins were expected to strongly influence viral protein synthesis. Since the unspliced viral RNA serves both as the mRNA for Gag and Gag-Pol synthesis and as the pregenome, we expected the levels of Gag and newly made virions to be strongly reduced by the SR proteins. To this end, levels of intracellular HIV Gag were assessed by CAp24 ELISA on cell lysates 48 h after DNA transfection (see methods). To measure the levels of virion production, culture supernatants were harvested every day for two days, pooled, clarified by filtration and ultracentrifuged through a 20 % sucrose cushion. Pelleted viral particles were resuspended in TNE buffer (see methods) and virus production was monitored by CAp24 ELISA. Series of measurements indicated that ASF/SF2 and SC35 caused about a 10–12 fold reduction of total Gag synthesized while 9G8 reduced it by roughly 4 fold. These results are in agreement with the relative levels of the unspliced viral RNA in HIV-1 producer cells (Table 1A).

**Figure 3:**
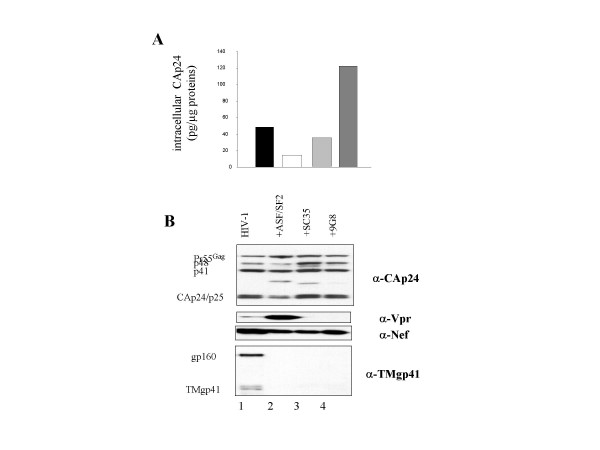
**Influence of SR proteins on HIV-1 protein synthesis**. 293T cells (2 × 10^5 ^per well) were transfected with 1 μg of HIV-1 pNL4.3 in the presence of increasing amounts of plasmid encoding either ASF/SF2, SC35 or 9G8. DNA concentrations were maintained constant by supplementation with the pCLacZ control plasmid which also served to monitor transfection efficiency. Values reported here correspond to assays carried out with a HIV to SR DNA molar ratio of 1:05. Cells were recovered two days after DNA transfection. A: Levels of Gag production were assessed by CAp24 antigen ELISA and expressed as pg of CA per μg of total cellular proteins. Note that ASF had a clear negative impact on Gag accumulation in cells whereas 9G8 had an opposite effect. B: Equivalent amounts of CAp24 antigen as measured by ELISA were subjected to western blotting. The same membrane was alternatively probed with the respective antibodies as indicated on the right: anti-CAp24 for Gag, anti-Vpr for p15, anti-NEF for p27 and anti-TMgp41 for Env. The viral Gag, Vpr, NEF and Env proteins are indicated according to their molecular weights in kDaltons. Note that SR proteins did not change the Gag processing pattern (compare lanes 2–4 and 1). ASF caused an indirect increase of Vpr cellular accumulation (lane 2) in agreement with its positive effect on Vpr mRNA level (Figure 1). On the other hand SC35 and 9G8 had an opposite effect (lanes 3–4). All Env levels were low (lanes 2–4) except in the control (lane 1).

Next we evaluated the relative amounts of cell-associated *versus *virion-associated Gag. Despite the low levels of total Gag synthesized as measured by CAp24 ELISA, cell-associated Gag was found at unexpected high levels when either one of the SR proteins was overexpressed. Indeed, cell-associated Gag levels were found to be about 40%, 80% and even 250% upon overexpressing ASF, SC35 and 9G8, respectively, as compared with control HIV-1 cells (Figure 3A). Pol was expressed as evidenced by Gag processing and the presence of reverse transcriptase (RT) in the newly formed infectious virions (see below). The pattern of Gag processing by the viral protease was only slightly influenced by overexpressing one of the SR proteins (Figure 3B, compare lanes 2–4 to 1, upper panel).

A large fraction of the 4 kb mRNAs codes for Env. The very low level of Env glycoproteins present in cells is consistent with the fact that SR proteins strongly reduced the encoding viral mRNA (Figure 2B; Figure 3B bottom panel).

Last we analysed viral protein synthesis directed by the multiple spliced 2 kb mRNAs, coding for the regulatory proteins Nef and Vpr and the trans-acting factors Tat and Rev. Only the expression of Vpr was found to be markedly enhanced by ASF/SF2 in agreement with the increased level of Vpr mRNAs (Figure 2; Figure 3B, compare lanes 1 and 2; and data not shown).

Thus we can conclude that the SR proteins have a strong indirect impact on viral protein synthesis due to their alterations of the HIV-1 splicing pattern. Only the rather high level of cell-associated Gag appears to contradict this view (see discussion).

### Influence of the SR proteins on Gag and Env expression analysed by immuno-confocal microscopy

**Figure 4:**
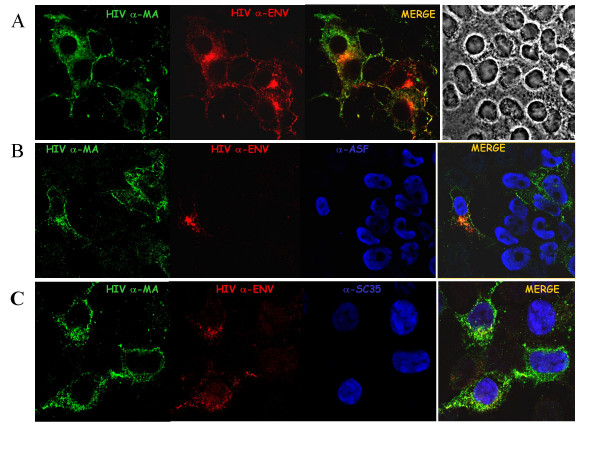
**Confocal microscopy of cells co-expressing HIV-1 Gag, Env and SR-protein**. Panel A: 293T cells expressing HIV-1 pNL4.3 were subjected to immuno-staining using anti-Map17 (green staining) and anti-Env gp120 (red staining) antibodies and staining was viewed by confocal microscopy as described in methods. Most if not all cells expressed Gag and Env but only partial colocalization was seen (merge picture). Right panel corresponds to the same cells viewed by phase contrast microscopy. Panel B: same as in A except that His tagged-ASF/SF2 SR protein was overexpressed by DNA transfection with about 75% transfection efficiency (see methods). ASF/SF2 protein is localized in the nucleus (blue staining) and its overexpression caused a drastic reduction of Env level while Gag remained well expressed in agreement with the western blot data (Figure 3) but with an heterogenous pattern (first panel). Panel C: same as in A except that His tagged-SC35 SR protein was overexpressed by DNA transfection with about 75% transfection efficiency (see methods). SC35 protein (nuclear blue staining) overexpression caused a reduction of Env level while Gag was still highly expressed in agreement with the western blot data (Figure 2). Note that in all cases examined here (anti-Map17; green staining in panel A to C) Gag was found to accumulate at the plasma membrane and in intracellular compartments corresponding to vesicles [42] (Muriaux *et al*., unpublished data).

**Figure 5:**
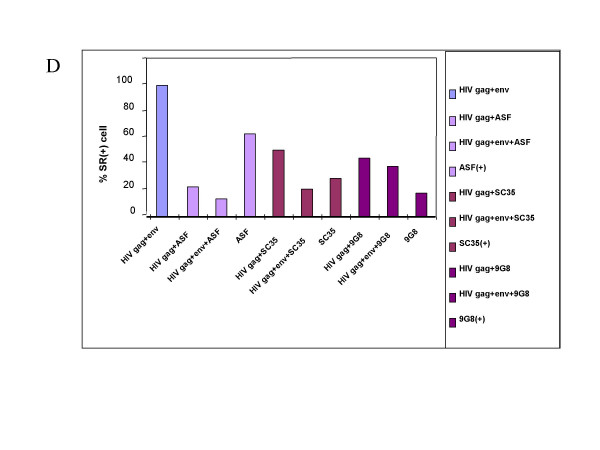
**Influence of SR protein on cellular levels of HIV-1 Gag and Env**. 293T cells expressing HIV-1 and one SR protein (either ASF, SC35 or 9G8) were immuno-stained, examined and counted using Confocal Laser Scanning Microscopy (see figure 4). Numbers are representative of more than 100 SR positive cells. For all experiments we evaluated the expression of Gag and Env, and SR protein when applicable. The numbers are expressed as the percentage of all SR positive cells given a DNA transfection efficiency of 70–75% (not shown). When HIV-1 pNL4.3 was transfected alone, 100% of the cells were found to co-express Gag and Env (first bar). Upon co-transfection with the ASF coding DNA, a majority of the cells only expressed ASF and about half of them expressed Gag and the SR protein (see ASF bars). Upon co-transfection of pNL4.3 and either the SC35 or 9G8 coding plasmid, a majority of cells expressed Gag and the SR protein (see SC35 and 9G8 bars).

To better understand the influence of the SR proteins on Gag and Env synthesis, we examined by immunofluorescence staining and confocal laser microscopy (CLSM), co-expression of the two major viral structural proteins in individual cells. HIV-1 expressing cells were subjected to immuno-staining using anti-MA for Gag (green staining) and anti-gp120 for Env (red staining) antibodies, and all stainings were viewed by confocal microscopy (Figure 4A) (see methods). It is noteworthy that most, if not all, cells co-expressed Gag and Env which accumulated at the plasma membrane and in intracellular vesicles (merge picture in Figure 4A). Co-expression of HIV-1 Gag and Env was confirmed by examining 100 cells where Gag only cells were hardly found, as expected with complete HIV-1 (Figure 5).

Overexpressing ASF/SF2 as evidenced by a blue nuclear staining in most cells (Figure 4B) caused a drastic reduction of Env but only moderately affected Gag (Figure 4B, green and red stainings) in agreement with the western blot data (Figure 3B, lane 2). As above, Gag was seen to accumulate in intracellular vesicles and at the plasma membrane while Env was expressed in a heterogeneous manner and mainly located in the cell interior (Figure 4B, HIV Env panel), probably in the Golgi area and in intracellular vesicles (Figure 4B, merge picture). Quantitative values on 100 cells, taking into account that 70–75% of the cells were positively transfected, showed that co-expression of Gag and ASF was observed in 25% of the cells while Gag, Env and ASF was seen in only 10% of the cells. At the same time 65% of the cells expressed ASF only (Figure 5, bars labelled ASF). These results further showed that the ASF/SF2 SR protein can have a drastic negative impact on HIV-1 since its overexpression caused a nearly complete suppression of Gag and Env expression in a large fraction of the cells (Figures 4B &5). SC35 (Figure 4C) and 9G8 (not shown) SR proteins had less pronounced effects since a majority of the cells coexpressed Gag and one SR protein (Figure 5; 45 to 55 % see bars labelled gag+SC35 and gag+9G8, respectively) or evenly in the case of Gag, Env and 9G8 (Figure 5; see bar labelled gag+env+9G8). These observations suggest that the SR proteins can have differential effects on HIV-1 structural protein expression.

The influence of the SR proteins on Gag and Env synthesis was further evaluated with respect to virion production and infectivity.

### Influence of SR proteins on virion production and infectivity

**Figure 6:**
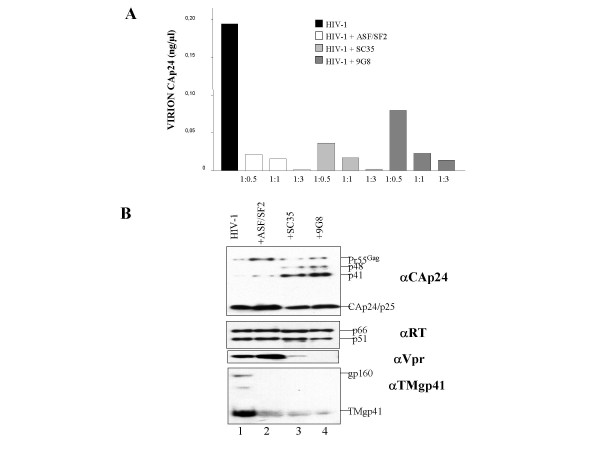
**Expression of viral proteins results from alterations of splicing pattern**. 293T cells (2 × 10^5 ^per well) were transfected with 1 μg of HIV-1 pNL4.3 in the presence of increasing amounts of plasmid encoding either ASF/SF2, SC35 or 9G8 (ratios indicate molar amounts of HIV-1 DNA *vs *SR-expressing vector). DNA concentrations were maintained constant by supplementation with the pCLacZ control plasmid which also served to monitor transfection efficiency. A: Viral production was monitored by CAp24 antigen ELISA and expressed as ng of p24 per ml of medium (see methods). Results are representative of 3 independent experiments. Note that the effect of ASF/SF2 on virion production was already drastic at a HIV/SR molar ratio of 1:0.5. B: The pelleted viral particles were tested for their content in Gag, Pol, Env and Vpr proteins. Equivalent amounts of CAp24 antigen measured by ELISA were subjected to Western blotting. The same membrane was alternatively probed with the respective antibodies as indicated on the right: anti-CAp24 for Gag, anti-RT for p66 and p51, anti-Vpr for p15 and anti-TMgp41 for Env. The viral Gag, RT, Vpr and Env proteins are indicated according to their molecular weights in kDaltons. Note that fully mature CAp24 and RTp66/p51 were abundant in all virion preparations. ASF caused an indirect increase of Vpr incorporation in virions (lane 2) whereas SC35 and 9G8 had an opposite effect (lanes 3–4). All Env levels were low (lanes 2–4) except in the control (lane 1).

This was examined by monitoring the levels of HIV-1 virion production under conditions of increasing expression of the SR proteins. As shown in Figure 6A, SR proteins overexpression induced a dose-dependent inhibition of virion production as compared with control cells co-transfected with HIV-1 pNL4.3 and an irrelevant expression vector. A high dose of SR DNA, notably ASF/SF2, caused a nearly complete inhibition of virion production.

Protein composition of the virions generated by cells overexpressing one of the SR factors, at a HIV-1/SR DNA ratio of 1:0.5, was investigated by western blotting using antibodies against the major core component, CAp24, the RT enzyme, viral factor VPR and the envelope glycoprotein TMgp41. As shown in Figure 6B, CAp24 and RT were found as processed Gag protein and Pol enzyme, respectively, in proportions similar or close to wt HIV-1 particles (see panels labelled αCAp24 and αRT). On the contrary, VPR was more abundant in virions upon overexpression of ASF/SF2 in agreement with higher levels of the corresponding viral mRNA and protein in cells (Figures 2 and 3B lane 2). With SC35 and 9G8 Vpr was hardly detected in virions in agreement with the very low level of Vpr mRNA and protein in cells (Figure 3B lanes 3–4). All SR proteins examined negatively impacted on the incorporation of Env TMgp41 in virions (Figure 6B, lanes 2–4), again in agreement with the fact that Env mRNA and protein levels were drastically reduced in cells (Figures 2 and 3B).

To test whether the decreased level of cellular unspliced viral RNA also caused an attenuation of genome packaging into newly made virions, viral particles corresponding to the same amounts of CAp24 were used to purify the genomic RNA which was analyzed by slot-blotting using a *gag*-specific probe. For all overexpressed SR proteins, genomic RNA packaging was reduced from 3 to 4 fold compared with control virions (Table 1B).

To determine the infectivity of virions produced by cells overexpressing one of the SR proteins, the same amount of virus-associated genomic RNA was used to infect Hela P4 cells, a HeLa subtype that constitutively expresses the CD4 receptor and contains the *lacZ *gene under the control of the HIV-1 LTR. One day later, blue cells were counted allowing us to assess virus infectivity (see methods). Upon overexpression of each one of the SR proteins, virus infectivity, based on the same amount of genomic RNA, was found to be 30 to 60% of the control virus, or 6 to 12 fold less based on identical amounts of CAp24-associated particles.

It can be concluded that overexpression of each one of the SR proteins caused a strong reduction of the unspliced viral RNA in cells, and this had a more pronounced effet on virion production than on Gag synthesis (Figures 2, 3, 4, 5, 6). At the same time levels of genomic RNA packaged into progeny virions remained high (Table 1). These findings are in full agreement with the fact that the genomic RNA is considered to be an indispensable partner of Gag in the course of virus assembly.

## Discussion

In the present study, we show that the overexpression of either one of three different SR proteins, namely ASF/SF2, SC35 and 9G8, profoundly affected the HIV-1 splicing pattern (Figure 1) [26], resulting in a drastic decrease of virus production. However, the progeny virions still made retained part of their infectivity. SR protein overexpression caused an oversplicing of the HIV-1 full length transcript and confirm that the targets of activation depend on the SR protein overexpressed. Indeed, ASF/SF2 stimulates splicing at sites A1 and A2, while SC35 and 9G8 preferentially enhance splicing at site A3 (Figures 1, 2). In addition to being general activators of constitutive splicing, results reported here confirm that each one of the three SR proteins exerts specific effects on the alternative splicing of HIV-1 primary RNA transcript (Figure 2) [26].

Little is known about the HIV-1 A1 site. Here, we show that ASF/SF2 participates in the utilization of A1 by a mechanism that requires further investigations. Many elements act in concert to repress splicing at site A2 such as its intrinsic weakness [5,6] and the existence of the hnRNP A/B dependent ESSV located in the noncoding exon flanking sites A2 and D3 [8,9]. Then how does ASF/SF2 exert this control ? The exon bridging hypothesis proposes that U1 snRNP binding to the downstream donor site acts to increase splicing efficiency at the upstream flanking acceptor site (for review see [20]). Also, SR proteins are known to stabilize U1 snRNP binding on suboptimal donor sites. Consequently, one can imagine that ASF/SF2 reinforces splicing at site A2 by stabilizing spliceosomal interactions at the suboptimal site D3. Accordingly, other SR proteins like SC35 or 9G8 would be expected to have the same effect as ASF/SF2 on site A2 and thus on Vpr mRNA synthesis. This prediction is inconsistent with our data (Figure 3) since overexpression of SC35 and 9G8 did not increase Vpr RNA level. Another possibility is that ASF/SF2 positively regulates splicing at site A2 by counteracting the effect of ESSV. ESSV represses splicing at site A2 by binding cellular hnRNP A/B proteins [8,9]. This binding prevents the assembly of U2AF on the polypyrimidine tract (PPT) and subsequently the formation of a functional spliceosome between sites D1 and A2 [9]. SR proteins are thought to also activate weak acceptor sites by facilitating the recruitment of U2AF on the PPT [20]. It is tempting to speculate that the ratio between hnRNP A/B and ASF/SF2 bound close to site A2 modulates the binding of U2AF at this site. This effect of SR proteins is generally mediated by a splicing enhancer, but whether an ASF/SF2-dependent splicing element is required here remains to be determined.

Site A3 is used to generate Tat mRNAs. Like A2, A3 is intrinsikly weak and repressed by hnRNP A/B-dependent ESS2 and hnRNP H-dependent ESS2p [17,29]. Our results show that a strong positive control is exerted by SR proteins SC35 and 9G8 at this level. Findings on SC35 are consistent with the recent data of Zahler *et al*. [19] reporting a novel ESE downstream of A3 that reinforces A3 utilization in the presence of a high level of SC35 *in vitro*. In addition, hnRNPs act in a *trans*-dominant manner to counteract that of SC35 *in vitro *[19]. Taken together these results strongly suggest that changing the hnRNP/SC35 ratio probably leads to activation or repression of splicing at site A3. Little is known on the implications of SR protein 9G8 in HIV-1 splicing. The present data show that 9G8 appears to function in a way similar to SC35 (Figure 2). As for ASF/SF2, the exon bridging hypothesis can be mentioned but 9G8 acts mainly by binding specific *cis*-acting RNA elements [30]. Even if overexpression of SC35 and 9G8 caused a large accumulation of Tat mRNAs, it is likely that these two SR proteins act by distinct mechanisms. Indeed, firstly SELEX experiments showed that 9G8 and SC35 recognize different consensus RNA sequences [30]. Secondly, ESS2 mutations that *in vitro *strongly reinforce the binding of SC35 on an ESS2-containing transcript have no effect on 9G8 binding to the same substrate [19,30]. Sequences important for 9G8 splicing activation remain to be determined.

In conclusion, the exact molecular mechanisms by which high levels of SR proteins cause a strong enhancement of genomic RNA splicing and consequently a severe inhibition of HIV-1 virion production remain to be determined. This is presently under investigation.

As expected, the profound changes of the HIV-1 splicing pattern caused by the overexpression of one of the SR proteins ASF/SF2, SC35 or 9G8 inhibited viral protein synthesis, notably that of the structural proteins Gag and Env (Figures 3, 4, 5) and consequently, virion production (Figure 6A). Still in agreement with such alterations of the HIV-1 splicing pattern (Figures 1, 2), Vpr synthesis was upregulated by ASF whereas SC35 and 9G8 had an opposite effect (Figure 3B). But it was surprising to find a rather high heterogeneous level of cell-associated Gag (Figure 3A). To explain this apparent discrepancy, one should remember that the unspliced viral RNA performs two essential functions, firstly as the mRNA for Gag and Gag-Pol synthesis and secondly as the pregenome for Gag assembly (reviewed in ref. [31]). In fact, the Gag assembly process requires two platforms that are the genomic RNA through specific NC-genomic RNA interactions [31,32] and a cellular membrane in which Gag is anchored *via *MA-membrane interactions (reviewed in ref. [33]). Membranes are not limiting whereas the full length RNA is probably limiting due to its mobilization by the translating ribosomes (reviewed in ref. [34]). Actually, the fate of the full length viral RNA appears to result from a subtle balance between Gag translation on ribosomes and core assembly governed by Gag-genomic RNA interactions (reviewed in refs [31,35]). In the presence of high amounts of the SR proteins, the unspliced viral RNA is even more limiting and thus probably rarely available for assembly. Hence, the cell-associated Gag corresponds to newly made free Gag molecules as well as Gag in newly assembled core nucleocomplexes which accumulate in the cell (Figure 4) before being released. According to this scheme of virus assembly, the low levels of the pregenomic RNA (Table 1A) and Env (Figure 3B) upon SR overexpression may very well explain why Gag assembly and virion release are most probably slowed down (Figure 6A). In agreement with this interpretation, the level of packaged genomic RNA into newly formed viral particles was decreased by 65 to 75% (Table 1B). Also in agreement with the above interpretation is the observation that production of high titer lentivectors necessitates expression of the recombinant viral RNA at high levels in vector producing cells [36].

Despite the low level of incorporated Env (Figure 6), virions produced by cells overexpressing one SR protein retained part of their infectivity on Hela P4 cells. This was not unexpected since only a minimal amount of Env appears to be required to drive infectivity in certain model cell systems [37].

## Conclusion

In summary, the data presented here show that elevated concentrations of SR proteins in HIV-1 expressing cells differentially affected viral RNA and protein expression, resulting in a strong decrease of viral progeny made. Thus one can speculate that the coordinated regulation of HIV-1 splice site utilization by SR proteins is of critical importance to maintain high levels and balanced ratios of the viral RNAs and hence of the viral proteins made in order to direct optimal virus assembly and production. Thus, HIV-1 probably needs to interact with the splicing machinery. In accordance with this view, SC35 is up-regulated and 9G8 down-regulated in HIV-1 infected cells [38,39]. On a more general basis it has been found that SR proteins influence expression and replication of other viruses such as human papilloma virus type 16 [40] and Adenovirus [41].

## Materials and methods

### Plasmids

Plasmids pXJ41-ASF, pXJ42-PR264 and pXJ42-9G8 [28] that respectively encode ASF/SF2, SC35 and 9G8 in eukaryotic cells were provided by J. Stevenin and R. Gattoni (IGBMC Strasbourg, France). The HIV-1 molecular clone is pNL4.3 (GenBank #M19921) [27].

### Cell cultures, transfections and infections

HeLa P4 (provided by P. Charneau) and 293T cells (provided by Genethon) were maintained in Dulbecco's modified Eagle's medium supplemented with 10 % fetal calf serum, 2 mM glutamine and antibiotics (penicillin-streptomycin; Invitrogen). One day before transfection, 3 × 10^6 ^293T cells were inoculated in 10-cm Petri dish (except for experiments of Figure 1, see legend). One day later, cells were transfected with 10 μg of proviral pNL4.3 and 4–10 μg of SR-expressing vector or of control plasmid pCLacZ by the calcium phosphate precipitation technique according to manufacturer instructions (Gibco). As observed by immuno-staining and confocal microscopy, more than 70% of the cells were positively transfected. After 12 h, cell culture supernatants were substituted by fresh culture medium. Forty-eight hours after transfection, supernatants were harvested, clarified by filtration through 0.8 μm-pore size filters and ultracentrifuged through a 20 % sucrose cushion. Pelleted viruses were resuspended in TNE buffer (25 mM Tris HCl pH 7.5; 150 mM NaCl; 1 mM EDTA). Virus production was monitored in cell culture supernatants and in virus pellets with a CAp24 ELISA capture assay (kindly provided by Valérie Cheynet and Bernard Mandrand, BioMérieux).

Viral titrations were performed by infection of HeLa P4 cells (1.5 × 10^5 ^cells per well of a 24-well plate) with purified viruses containing 1 ng of genomic RNA. After 24 h, cells were fixed and incubated in the presence of X Gal substrate at 37°C until blue color development was complete. Viral titers were determined by counting the number of blue cells in threee different wells.

### RNA isolation, Northern blotting and RT-PCR

Transfected cells were harvested 48 h after transfection and washed in phosphate-buffered saline (PBS). Two-third of cells were resuspended in PBS and total cellular RNA was extracted with TRIzol reagent as recommended by the manufacturer (Invitrogen). Culture supernatants were treated as indicated above and the level of CAp24 antigen measured by ELISA. Northern blotting of intracellular viral RNAs was performed with 10 μg of total RNA and slot-blot of virion-associated genomic RNA was performed with 10 ng of CAp24 antigen. After transfer onto a Hybond-N+ membrane (Amersham Pharmacia Biotech), viral RNAs were probed with radiolabeled fragments from Env for Northern blot and from Gag region for slot-blot. All the mRNAs species were quantified by Storm (Amersham).

The splicing products were analyzed by RT-PCR as previously described [10], except that the forward PCR primer was Odp045 [2]. The PCR products were fractionated on a 6 % acrylamide-7M urea electrophoresis gel and autoradiographed. Individual HIV-1 mRNA species were named according to the nomenclature of Purcell and Martin [2].

### Immunoblotting

One-third of transfected cells were washed in PBS and lysed in PBS containing 0.5 % Triton. After CAp24 ELISA measurement in cell lysates and in virus pellets, samples were added to 3X gel loading buffer (0.5 M Tris-HCl pH 6.8; 0.8 % SDS; 40 % glycerol; 5 % β-mercaptoethanol; 0.03 % bromophenol blue). For immunoblotting, samples containing equal amounts of CAp24 antigen were loaded on a 10 % SDS-PAGE and fractionated proteins were transferred onto a Hybond P membrane (Amersham Pharmacia Biotech). Viral proteins were probed with monoclonal anti-CAp24 (BioMérieux), polyclonal anti-Vpr (#3951, NIH USA), polyclonal anti-Nef (#331, NIH USA) or monoclonal anti-TMgp41 (Ab 41A9; Hybridolab, Pasteur) antibodies. The bound antibodies were detected with peroxidase-conjugated anti-mouse IgG antibodies and visualized by the SuperSignal West Pico Chemiluminescent Substrate (Pierce).

### Immunofluorescence staining and confocal microscopy imaging

Transfected 293 T cells were grown on poly-lysine coated coverglass dishes and fixed 24 h post transfection in 3% paraformaldehyde (diluted in Phosphate Buffer Saline – PBS) for 20 min. The fixative was then removed and the free aldehydes were quenched with 50 mM NH_4_Cl. Cells were then permeabilized using 0.2 % Triton X-100 for 5 min and blocked in 1% BSA. The fixed cells were incubated for one hour at room temperature with primary antibodies: rabbit anti-MAp17 (NIH, USA), human anti-HIV-1 gp120 Mab(b12) (NIH, USA), rabbit anti-His for His-tagged 9G8 and ASF proteins, and mouse anti-HA1 for HA-tagged SC35 protein (Sigma). The corresponding fluorescent Alexa^® ^488, 546 and 633-conjugated secondary antibodies were used at 0.5 μg/ml (Molecular probes). Coverslips were washed three times with PBS and mounted on microscope slides with Mowiol (Sigma). Images were acquired on Axioplan 2 Zeiss CLSM 510 confocal microscope with Argon 488/458, HeNe 543, HeNe 633 lasers and plan apochromat 63 × 1.4 oil Ph3 objective, supplied with LSM 510 3.4 software.

## Abbreviations used

HIV-1, Human Immunodeficiency Virus type 1.

CAp24, viral capsid protein p24.

MAp17, viral matrix protein p17.

RT, reverse transcriptase.

SR, splicing regulatory proteins.

hnRNP proteins, heterogenous ribonucleoparticle proteins.

Site A, splicing acceptor site. Site D, splicing donor site.

Kb, kilobases. WT, wild type.

PBS, Phosphate Buffer Saline.

BSA, bovine serum albumin.

Mab, monoclonal antibody.

## Authors' contributions

SJ carried out analyses on SR protein-mediated effect on HIV-1 RNA splicing.

DD was in charge of cell culture and transfection assays. DM performed immuno-confocal microscopy experiments and analyses. JLD is the lab head and arranged the manuscript.

## Competing interests

The author(s) declare that they have no competing interests.
